# Derivation of a no‐significant‐risk‐level for tetrabromobisphenol A based on a threshold non‐mutagenic cancer mode of action

**DOI:** 10.1002/jat.3594

**Published:** 2018-02-13

**Authors:** Alison M. Pecquet, Jeanelle M. Martinez, Melissa Vincent, Neeraja Erraguntla, Michael Dourson

**Affiliations:** ^1^ Risk Science Center, Department of Environmental Health, College of Medicine University of Cincinnati 160 Panzeca Way Cincinnati OH 45213 USA; ^2^ American Chemistry Council 700 2nd St NE Washington DC 20002 USA

**Keywords:** benchmark dose, cancer threshold, CAS RN 79‐94‐7, mode of action, NSRL, RfD_cancer_, risk characterization, TBBPA, tetrabromobisphenol A, uterine cancer

## Abstract

A no‐significant‐risk‐level of 20 mg day^–1^ was derived for tetrabromobisphenol A (TBBPA). Uterine tumors (adenomas, adenocarcinomas, and malignant mixed Müllerian) observed in female Wistar Han rats from a National Toxicology Program 2‐year cancer bioassay were identified as the critical effect. Studies suggest that TBBPA is acting through a non‐mutagenic mode of action. Thus, the most appropriate approach to derivation of a cancer risk value based on US Environmental Protection Agency guidelines is a threshold approach, akin to a cancer safe dose (RfD_cancer_). Using the National Toxicology Program data, we utilized Benchmark dose software to derive a benchmark dose lower limit (BMDL_10_) as the point of departure (POD) of 103 mg kg^–1^ day^–1^. The POD was adjusted to a human equivalent dose of 25.6 mg kg^–1^ day^–1^ using allometric scaling. We applied a composite adjustment factor of 100 to the POD to derive an RfD_cancer_ of 0.26 mg kg^–1^ day^–1^. Based on a human body weight of 70 kg, the RfD_cancer_ was adjusted to a no‐significant‐risk‐level of 20 mg day^–1^. This was compared to other available non‐cancer and cancer risk values, and aligns well with our understanding of the underlying biology based on the toxicology data. Overall, the weight of evidence from animal studies indicates that TBBPA has low toxicity and suggests that high doses over long exposure durations are needed to induce uterine tumor formation. Future research needs include a thorough and detailed vetting of the proposed adverse outcome pathway, including further support for key events leading to uterine tumor formation and a quantitative weight of evidence analysis.

## Abstract 2

A non‐mutagenic threshold mode of action for tetrabromobisphenol A was utilized to derive a no‐significant‐risk‐level of 20 mg day^–1^ for uterine tumors (adenomas, adenocarcinomas, and malignant mixed Müllerian combined) observed in female Wistar Han rats from a National Toxicology Program 2‐year bioassay. The most recent available techniques were utilized, including literature review, benchmark dose software, mode of action analysis, and threshold extrapolation. The derived no‐significant‐risk‐level aligns well with other available non‐cancer and cancer risk values based on the biology of these tumors.

## 1 INTRODUCTION

Under the State of California's Proposition 65 (Prop65), a no‐significant‐risk‐level (NSRL) is developed for chemicals that the State views as “known to cause cancer.” The NSRL represents the “levels of exposure calculated to result in no more than one excess case of cancer in an exposed population of 100,000, assuming exposure over a 70‐year lifetime (10^–5^ lifetime risk of cancer)” (Office of Environmental Health Hazard Assessment, OEHHA, 1989). California's OEHHA recently announced its Prop65 notice of intent to list tetrabromobisphenol A (TBBPA) as known to the state to cause cancer. This is likely based on a recent International Agency for Research on Cancer (IARC) assessment that classified TBBPA as “Group 2A: probably carcinogenic to humans” (IARC Monograph in preparation, Volume 115 – only the classification is available at the time of publication; Grosse et al., 2016). With the addition of TBBPA to Prop65, a toxicological evaluation of TBBPA and derivation of an NSRL is needed.

The methodology for NSRL derivation (OEHHA, 1989) is similar to that of the US EPA (2005) for developing cancer potency values. An evaluation of the available toxicological data in humans and animals is used to identify a significant biologic response of concern, also referred to as the critical effect. In the absence of data to the contrary, no threshold is assumed for the cancer effect of concern, and OEHHA (2013) then develops an NSRL through the use of no‐threshold models (cancer slope factor development) based on US EPA guidance (1986, 2005). These NSRL values are then compared to exposure estimates to determine the potential to evoke a biological response at relevant environmental exposure levels. If the exposure estimates are at or lower than the NSRL, then the exposure to the population is considered acceptable within a margin of safety (OEHHA, 1989). However, when a threshold in response is supported based on available data, many risk agencies around the world support alternative approaches such as using threshold models. For example, the US EPA (2005) methodology has advanced the state of risk science, and includes a determination of a linear (non‐threshold) or non‐linear (threshold) mode of action (MOA) approach. The European Food Safety Authority (EFSA) and European Chemicals Agency (ECHA), among other regulatory bodies, also recognize biological thresholds in their assessments (Bevan & Harrison, 2017). Threshold models suggest that there are low doses of a chemical that do not cause effects and that a high enough dose is needed above this threshold for effects to occur, while non‐threshold models suggest that any dose above 0 can lead to an effect (US EPA, 2005).

One basis for the non‐threshold models relates to mutagenic chemicals that cause DNA damage, which in turn contributes to carcinogenesis, regardless of dose. In fact, identification of mutagenicity mechanisms for cancer development is often a key diagnostic for identification of threshold vs. non‐threshold mechanisms (Bevan & Harrison, 2017). This determination affects the choice of either the derivation of a cancer slope factor and a risk‐specific dose, or a threshold‐based toxicity reference value for cancer effects (RfD_cancer_). Accordingly, two recent NSRLs were developed for diethanolamine (Kirman, Hughes, Becker, & Hays, 2016) and titanium dioxide (Thompson et al., 2016) using threshold approaches based on non‐mutagenic MOAs.

TBBPA, a flame‐retardant chemical that is detected in the environment, albeit at low levels in the USA, has been extensively studied for a number of years. To develop an NSRL, we first reviewed available assessments for TBBPA from regulatory and other agencies to see if an extant cancer risk value had been derived that could be adapted for use. A literature search was conducted from the date of the most recent regulatory review to the present to identify any new data published since the time of the last review that could inform or update the basis for the NSRL. Data from both the reviews and the published literature were evaluated for toxicological data and MOA information pertinent to cancer development. A risk characterization was then conducted, building off of previous publications, by identification of the critical tumor effect, identification of a point of departure (POD) utilizing benchmark dose (BMD) modeling, review of the MOA for tumor formation, derivation of a cancer risk value, and adaptation to an NSRL.

## 2 METHODS

### 2.1 Literature search and hazard identification

There are a number of comprehensive reviews available from regulatory agencies and others summarizing the toxicology and potential health impacts from exposure to TBBPA. These were identified through an Internet search in relevant regulatory databases. The Internet was searched by individual key agency web sites and broadly with ToxPlanet (https://toxplanet.com/). Additionally, an updated literature search was conducted from a few years before the date of the most recent review document (Health Canada, 2013), to identify any newly published data that could be utilized in the derivation of the NSRL.

The literature used in this report was in part identified in a systematic literature search in Elsevier Embase, PubMed, and ToxPlanet databases conducted in September 2016 for the previous 5 years (2011–16). The results and details of these searches can be found in Table 1. A broad ranging search in each database was initially utilized by searching the chemical name, synonyms, CAS registry number, and relevant acronyms. Data were filtered by limiting to animal or human species. In PubMed, another filter was employed – “NOT prealbumin” – as this key word was not relevant to toxicology studies but appeared repeatedly in the search results. Identified literature was initially screened and reviewed by title and abstract for content and relevance, and selected literature was subsequently obtained and further reviewed for appropriate data. These studies were reviewed and evaluated to determine the most appropriate critical cancer effect for use in deriving the NSRL. Literature regarded as insufficiently reliable for supporting a health conclusion (e.g., inadequate description of methods or data, lack of appropriate dose–response data) were excluded from further consideration.

**Table 1:** Detailed search terms, search strings and resulting number of hits for each database searched to identify literature for use in derivation of the TBBPA NSRL

Database	Search string	No. of hits
PubMed	Tetrabromidiphenylolpropane OR tetrabromodi OR tetrabromodi) OR tetrabromobisphenol OR Tetrabromo‐4,4′‐isopropylidenediphenol OR fire guard 2000 OR 79‐94‐7 OR tbbpa OR 3,5,3′,5′‐Tetrabromobisphenol A	6994
PubMed	Added NOT “prealbumin”	863
PubMedLAST 5 YRS	tetrabromidiphenylolpropane OR tetrabromodi OR tetrabromodi OR tetrabromobisphenol OR Tetrabromo‐4,4′‐isopropylidenediphenol OR fire guard 2000 OR 79‐94‐7 OR tbbpa OR 3,5,3′,5′‐Tetrabromobisphenol A Filter: published in the last 5 years; Animals	135
PubMedLAST 5 YRS	tetrabromidiphenylolpropane OR tetrabromodi OR tetrabromobisphenol a OR Tetrabromo‐4,4′‐isopropylidenediphenol OR “Great Lakes BA‐59P” OR “BA 59” OR 4,4′‐Isopropylidenebis 2,6‐dibromophenol OR 3,5,3′,5′‐Tetrabromobisphenol A OR 2,2′,6,6′‐Tetrabromobisphenol A OR 79‐94‐7 OR tbbpa AND “last 5 years”[PDat] NOT PREALBUMIN Filters: published in the last 5 years; Humans	78
EMBASE	tetrabromidiphenylolpropane OR tetrabromodi OR tetrabromobisphenol a OR “tetrabromo 4 4 isopropylidenediphenol” OR 4 4 isopropylidenebis (2,6‐dibromophenol) OR 3 5 3 5 tetrabromobisphenol a OR 2 2 6 6 Tetrabromobisphenol A OR 79‐94‐7 OR tbbpa	751
EMBASE	ABOVE (TBBPA STRING) AND animal experiment OR animal tissue OR controlled study OR correlational study OR human OR in vivo study OR intermethod comparison OR nonhuman OR normal human OR validation process OR validation study AND (2011:py OR 2012:py OR 2013:py OR 2014:py OR 2015:py OR 2016:py OR 2017:py)	316
ToxPlanet	TBBPA; 79‐94‐7	91

As detailed below, due to the lack of available cancer studies other than the US National Toxicology Program (NTP) 2014, 2‐year cancer bioassay, NTP (2014) was chosen for use in the identification of the critical effect. Additional review papers and published literature (described below) were evaluated to gain an understanding of the non‐cancer effects of TBBPA as well as the potential MOA for tumor formation.

### 2.2 Dose–response analysis to derive point of departure

BMD modeling (BMDS 2.6; US EPA, 2012) was used to evaluate the dose–response relationship between exposure to TBBPA and cancer outcomes. As detailed below, adenoma, adenocarcinoma, or malignant mixed Müllerian tumors (MMMTs) (combined) of the uterus identified through both original and residual longitudinal reviews (see Table 2 in Dunnick et al., 2015; NTP, 2014) were modeled to identify a POD. Atypical hyperplasia of the endometrium was also considered (see Table 6 in NTP, 2014). All standard dichotomous models were evaluated. BMDs corresponding to 10% extra risk, the benchmark response (BMR), and their 95% lower bounds (BMDLs) were determined. All BMD modeling was done using extra risk. Model parameters were restricted when possible; not all models offer an option for the restriction of the slope or power. The POD reported is the duration‐adjusted dose (i.e., the dose × 5/7, to account for dosing on only 5 of 7 days per week).

**Table 2:** TRVs identified in the literature for the general population and breakdown of how each value was derived

Reference	TRV	Value (mg kg^–1^ day^–1^)	Exposure duration, route	Critical effect	Key study	Point of departure	Composite adjustment factor (individual adjustments)
ECHA (2017)	DNEL	2.5	Chronic, oral	Unidentified; however, the registration dossier states “a chronic study is used to set a chronic DNEL. No correction required”	Mice, oral gavage (study citation not clear)	NOAEL = 250 mg kg^–1^ day^–1^	100 (UF_A_ = 10, UF_H_ = 10)
Colnot et al. (2014)	DNEL	5	Chronic, oral	No reproductive/developmental effects	Rats, oral gavage (MPI Research, 2002b, cited in Colnot et al., 2014)	NOAEL = 1000 mg kg^–1^ day^–1^	200 (UF_A_ = 10, UF_H_ = 10, UF_S_ = 2)
Colnot et al. (2014)	DNEL	0.16	Chronic, oral	Thyroid hormone changes	Rats, dietary (Van der Ven et al., 2008, cited in Health Canada, 2013 and EFSA, 2011)	BMDL_10_ = 16 mg kg^–1^ day^–1^	100 (UF_A_ = 10, UF_H_ = 10)
Colnot et al. (2014)	DNEL	10	Chronic, oral	No reproductive/fertility effects	Rats, oral gavage (MPI Research, 2001, cited in Colnot et al., 2014)	NOAEL = 1000 mg kg^–1^ day^–1^	100 (UF_A_ = 10, UF_H_ = 10)
COT (2004)	TDI	1	Chronic, oral	No embryotoxic/teratogenic effects	Rats, oral gavage (MPI, 2002b, cited in Colnot et al., 2014)	NOAEL of 1000 mg kg^–1^ day^–1^	1000 (UF_A_ = 10, UF_H_ = 10, UF_D_ = 10)
Wikoff et al. (2015)	RfD	0.6	Chronic, oral	Uterine endometrial atypical hyperplasia	Rats, oral gavage (NTP, 2014)	BMDL_10_ = 72.8 mg kg^–1^ day^–1^ HED = 18.2 mg kg^–1^ day^–1^	30 (UF_A_ = 3; UF_H_ = 10) NSRL for cancer precursor effect for 70 kg human = 42 mg kg^–1^ day^–1^
Pecquet et al. (2017, this paper)	RfDcancer	0.3	Chronic, oral	Uterine tumors	Rats, oral gavage (NTP, 2014)	BMDL_10_ = 102.5 mg kg^–1^ day^–1^ HED = 25.6 mg kg^–1^ day^–1^	100 (UF_A_ = 3; UF_H_ = 10; UF_D_ = 3) NSRL for 70 kg human = 20 mg kg^–1^ day^–1^
Wikoff et al. (2015)	Cancer slope factor	0.00315	Chronic, oral	Uterine tumors	Rats, oral gavage (NTP, 2014)	BMDL_10_ = 126.6 mg kg^–1^ day^–1^ HED = 31.7 mg kg^–1^ day^–1^	RSD at 10^–6^ = 0.0032 mg kg^–1^ day^–1^ NSRL for 70 kg human = 0.22 mg kg^–1^ day^–1^

BMDL_10_, benchmark dose lower limit; DNEL, derived no effect level; HED, human equivalent dose; NOAEL, no‐observed‐adverse‐effect‐level; NSRL, no‐significant‐risk‐level; RSD, risk‐specific dose; TDI, tolerable daily intake; TRV, toxicity reference value.

The EFSA (2017) BMD modeling criteria suggests the Akaike information criterion (AIC) to assess model fit. US EPA's BMDS guidance document for interpreting modeling results recommends adequacy determinations based on *P* value, scaled residuals, visual fit, consideration of variability among BMDLs across the candidate models, AIC, and professional judgment (US EPA, 2012). We have briefly covered our decision criteria below. Further information on these criteria can be found in the available guidance documents (EFSA, 2017; US EPA, 2012).

The first criterion is the global statistical goodness of fit test that represents the full dose range of the data. If *P* > 0.1, then the model is considered to fit the data adequately. Values lower than 1 suggest that the model may be statistically significantly different from the data, with values of 0.05 or less decidedly so. Models with *P* < 0.1 are usually rejected. However, models with higher *P* values are not necessarily better than models with lower *P* values (e.g., *P* = 0.5 vs. *P* = 0.2) if both have *P* > 0.1, which is why other criteria, described below, are then used.

The second criterion is the difference in scaled residuals (i.e., the difference in the modeled estimate compared with the actual data scaled by the standard error) at the data point closest to the BMR (in this case, 10%), where it is most important that the model fits the data. A scaled residual with an absolute value of less than 2 is acceptable.

The third criterion, related to scaled residuals, is the visual fit. Arguably, the least quantitative criterion, visual fit nevertheless allows consideration of how well the model fits the underlying data, particularly at the lower end of the curve, or how well the model reflects the biological MOA, if known. Designations of visual fit can include good, acceptable, and poor.

The fourth criterion is twofold. The first part asks whether the BMDL estimates from the remaining models are sufficiently close to each other and reflect no particular influence of the individual models. This emphasizes that the goal of the modeling is to calculate a BMDL. One way to view this is to compare the ratios between the BMD and BMDL among the models. The larger the ratio, the less accurate the model is likely to be.

The second part of this fourth criterion is the AIC. Of the remaining models, the one chosen will generally have the lowest AIC. However, AICs within a factor of 2 of each other are considered similar.

### 2.3 Derivation of no‐significant‐risk‐level

Once the POD was derived using BMDS, standard risk assessment guidance was utilized for the derivation of a cancer risk value and adaptation to an NSRL based on the US EPA (2005) and OEHHA (1989) methodology. We first adjusted the POD to a human equivalent dose (HED) using allometric scaling (Equation (1)). Because the weight of evidence for MOA for tumor formation identified did not involve direct DNA interaction, traditional linear cancer slope factor derivation was not conducted (NTP, 2014; Wikoff et al., 2015; Wikoff, Rager, Haws, & Borghoff, 2016). Instead, an RfD_cancer_ was derived for a non‐linear threshold response following the guidance of US EPA (2005). This includes an assessment of the uncertainty associated with the POD and the application of uncertainty factors (UFs; Equation (2)). UFs are used to add conservatism and additional safety to the RfD_cancer_ given unknowns about the chemical and to account for data gaps, such as animal to human uncertainty, subchronic to chronic exposures, and to account for intra‐individual variability. The derived RfD_cancer_ was then converted to an NSRL by adjusting for body weight (Equation (3)).

(1) $${\text{Dose}}_{H}={\text{Dose}}_{A}\times {\left({\mathrm{BW}}_{A}/{\mathrm{BW}}_{H}\right)}^{1/4}$$

Where

Dose_H_: dose in human (BMDL_10[HED]_)
Dose_A_: dose in animal (the POD for the specified critical effect = BMDL_10_)
BW_A_: body weight of animal (0.268 kg for control female Wistar from NTP)
BW_H_: body weight of human (70 kg)

(The body weight of 70 kg is the default body weight for males used by OEHHA as listed in the California Code of Regulations [27 CCR §25703, 27 CA ADC §25703; OEHHA, 2013]. However, the recommended body weight for females is 58 kg, which is the specific subpopulation of interest for this tumor type, as uterine tumors were identified as the critical effect and will only occur in females. We chose to use the 70 kg default as the body weight because: [1] it is more conservative [results in a slightly lower HED] than 58 kg; [2] women in the USA tend to be heavier; [3] 70 kg was utilized in most of the previous NSRL documents that we reviewed; and [4] due to the nature of the assessment, the difference between 70 and 58 kg is not enough to change significantly the final NSRL value [within an order of magnitude].)

(2) $${\mathrm{RfD}}_{\text{cancer}}={\text{BMDL}}_{10\left[\mathrm{HED}\right]}/\left({\mathrm{UF}}_{H}\times {\mathrm{UF}}_{A}\times {\mathrm{UF}}_{S}\times {\mathrm{UF}}_{L}\times {\mathrm{UF}}_{D}\right)$$

Where

BMDL_10[HED]_: BMD lower limit HED
UF_H_: UF for human variability
UF_A_: UF for animal to human extrapolation
UF_S_: UF for subchronic to chronic extrapolation
UF_L_: UF for lowest observed adverse effect level to no‐observed‐adverse‐effect‐level (NOAEL)
UF_D_: UF for database completeness

(3) $$\text{NSRL}\;\left(\mathrm{mg}/\mathrm{day}\right)={\mathrm{RfD}}_{\text{cancer}}\left(\mathrm{mg}\;{\mathrm{kg}}^{–1}{\mathrm{day}}^{–1}\right)\times {\mathrm{BW}}_{H}\left(\mathrm{kg}\right)$$

Where,

BW_H_: body weight of human (70 kg).

## 3 RESULTS

### 3.1 Literature search results

Comprehensive reviews identified include the National Institute of Environmental Health Sciences (NIEHS, 2002), the European Union (EU, 2006), the European Commission Committee on Toxicology (COT, 2004), the EFSA (2011), and Health Canada (2013). At the time of this publication, the IARC monograph on TBBPA was unavailable for public review, and only the classification was available (Grosse et al., 2016). The above‐mentioned and available regulatory toxicity reference values for cancer (and non‐cancer) effects for TBBPA were evaluated. However, of these reviews, only two oral toxicity reference values were derived (COT, 2004; ECHA, 2017). Our literature search identified three additional recently published papers that derived risk values for TBBPA (Colnot, Kacew, & DeKant, 2014; Wikoff et al., 2015; Yang, Ni, Yu, Cai, & Yu, 2016). All values were evaluated for relevance in adapting for use as the NSRL. Data were also mined from the two most recent regulatory reports (EFSA, 2011; Health Canada, 2013) relating to standard toxicological endpoints and agency conclusions on the potential for adverse health effects in humans. All publically available data were reviewed, synthesized and, in the absence of an available cancer risk value for TBBPA from the regulatory agencies, a cancer risk value was derived and the OEHHA methodology was applied to translate this value into an NSRL. (Note that ECHA is not considered an “authoritative review.” ECHA only disseminates industry data sets without reviewing the science content systematically, and does not include the complete submission. The rationale for the derived no effect level [DNEL] is in the original submission to ECHA, but not publicly disseminated because of intellectual property rights.)

The literature search identified a carcinogenicity study of TBBPA by the US NTP (NTP, 2014), and associated published studies that evaluated these NTP (2014) tumor findings and the TBBPA cancer MOA (Dunnick et al., 2015; Hall, Coulter, Knudsen, Sanders, & Birnbaum, 2017; Harvey et al., 2015; Lai, Kacew, & Dekant, 2015; Sanders et al., 2016; Wikoff et al., 2015; Wikoff et al., 2016). These data are pertinent because the lack of cancer data was identified as a data gap precluding the development of a cancer potency value by regulatory agencies (EFSA, 2011; Health Canada, 2013). Further studies were identified investigating non‐cancer effects related to inhalation toxicity, dermal absorption, thyroid hormone disruption, endocrine activity, developmental toxicity, and neurotoxicity. Additional toxicokinetic studies that were identified reported the disposition and kinetics of TBBPA in rats, and one investigated toxicokinetic parameters in humans.

### 3.2 Regulatory and published risk values for tetrabromobisphenol A

#### 3.2.1 Toxicity reference values

Toxicity reference values for TBBPA from various agencies are summarized in Table 2. The UK COT (2004) derived a tolerable daily intake (TDI) for oral exposure of 1 mg kg^–1^ day^–1^ for chronic exposure in the general population. This TDI was based upon a NOAEL of 1000 mg kg^–1^ day^–1^ in an unpublished two‐generation reproductive toxicity study and in an unpublished 90 day study (MPI Research, 2002a,b, as cited in COT, 2004). The COT applied a composite UF of 1000 based on 10 for human to animal (UF_A_), 10 for human variability (UF_H_) and 10 for database deficiencies (UF_D_).

ECHA (2017) reported a DNEL for long‐term systemic effects following oral exposure for the general population. The oral DNEL of 2.5 mg kg^–1^ day^–1^ available on the ECHA website does not provide enough publically available detail to determine the NOAEL used or the UFs applied to derive the value. (This information is available in the chemical safety report and fields that are not disseminated publicly, but can be obtained on request by the Lead Registrant and ECHA. However, we did not request this information.) Colnot et al. (2014) reported four oral DNELs, two based on different endpoints (thyroid effects and no effect in a 90 day study) and two for reproductive endpoints (fertility and development). The lowest oral DNEL of 0.16 mg kg^–1^ day^–1^ was based on a BMDL_10_ of 16 mg kg^–1^ day^–1^ for thyroid hormone changes after application of a 100‐fold UF (UF_A_ = 10, UF_H_ = 10).

Two recently published reference values for TBBPA were identified in the literature search (Wikoff et al., 2015; Yang et al., 2016) (Table 2). Yang et al. (2016) compared previous PODs available in the literature for TBBPA with a POD generated in their own study investigating TBBPA toxicity to thyroid hormones. However, due to a lack of some methodological details in the publication, the Yang et al. (2016) assessment was not used in supporting the derivation of a cancer risk value. For example, the authors do not discuss the UFs used to derive the RfD, the details of the BMD model outputs, or rationale for model choice. Without these methodological details, there is not enough information provided to analyze the proposed RfD.

In the other assessment, Wikoff et al. (2015) developed a number of non‐cancer and cancer toxicity reference values, including an oral RfD, oral cancer slope factor, average daily dose estimate, and evaluated the margin of exposure (MOE) and margin of safety based on these risk values. These toxicity reference values were based on the recent NTP 2‐year bioassay in rats and mice (NTP, 2014) and followed standard US EPA methodology, including the use of BMD modeling (US EPA, 2012). Wikoff et al. (2015) conducted a comprehensive literature search to identify published and unpublished TBBPA toxicity studies. Their search identified a data set of studies to review, and was followed by an evaluation of study quality using Klimisch scoring that narrowed the database to the most relevant high‐quality studies (Klimisch, Andreae, & Tillmann, 1997). The authors then selected the NTP (2014) 2‐year carcinogenicity assay from the high‐quality studies and identified the most sensitive cancer and non‐cancer endpoints for their choice of PODs (Wikoff et al., 2015).

For the non‐cancer RfD, Wikoff et al. (2015) selected female rat uterine hyperplasia from the 2‐year NTP bioassay as the critical effect. The data were modeled using BMDS to derive a BMDL_10_ of 72.8 mg kg^–1^ day^–1^ and, after adjustment for allometric scaling to humans, resulted in a HED of 18.2 mg kg^–1^ day^–1^. Using this POD, a composite UF of 30 was applied (UF_A_ = 3, UF_H_ = 10) resulting in an RfD of 0.6 mg kg^–1^ day^–1^. It is worth noting that the BMD model applied (unspecified in the publication) had poor fit (*P* = 0.08) even after dropping the high‐treatment dose (Wikoff et al., 2015).

For cancer endpoints, Wikoff et al. (2015) considered uterine tumors from the NTP (2014) study as the most appropriate endpoint for use in the derivation of a cancer toxicity value. Wikoff et al. (2015) applied the linear multistage BMD model to the duration‐adjusted doses for the cancer data set. Their BMDL_10_ was 127 mg kg^–1^ day^–1^, and after adjustment for allometric scaling to humans, resulted in an HED of 31.7 mg kg^–1^ day^–1^. Using this POD, the cancer slope factor was calculated to be 0.0032 mg kg^–1^ day^–1^, which corresponds to a risk‐specific dose at the 10^–5^ level of 0.0032 mg kg^–1^ day^–1^ (Wikoff et al., 2015). This value has been through a quality assurance review and is posted on the International Toxicity Estimates for Risk database, which is found on the US National Library of Medicine's TOXNET (https://www.nlm.nih.gov/pubs/factsheets/toxnetfs.html). Of note, the oral slope factor was likely derived due to the lack of robust MOA data needed to move away from the linear extrapolation default, based on regulatory guidance.

Of all the studies reviewed, only the Wikoff et al. (2015) characterized the cancer human health risks of exposure to TBBPA by developing cancer potency values (Table 2). Several organizations concluded that there were not sufficient data available to derive cancer toxicity reference values (as the assessments were concluded before publication of the NTP report), and many applied an MOE approach. An MOE can be defined as the magnitude by which the POD (e.g., the NOAEL) of the most sensitive relevant toxic effect exceeds the estimated exposure (Barnes & Dourson, 1988).

### 3.3 Summary of tetrabromobisphenol A toxicology

To understand the potential for toxicity from TBBPA exposure, the non‐cancer and cancer toxicity findings from recent regulatory agencies were reviewed. Overall, TBBPA is expected to have a very low systemic non‐cancer toxicity, with low hazard for developmental or reproductive toxicity, as reviewed and reported in multiple regulatory and other published reports (Colnot et al., 2014; Cope, Kacew, & Dourson, 2015; ECHA, 2017; EFSA, 2011; Health Canada, 2013; NTP, 2014; US EPA, 2014; etc.).

#### 3.3.1 Genotoxicity and cancer

EFSA (2011) found no in vivo studies available to assess the genotoxicity of TBBPA, and Health Canada (2013) identified no structural activity data suggesting TBBPA might be genotoxic. Further, a number of in vitro studies, such as several Ames tests and mutagenicity assays, a chromosomal aberration assay, a recombination assay, a sister chromatid exchange in Chinese hamster ovary cells, and a rat hepatocyte unscheduled DNA synthesis assay were evaluated, all with negative findings (Colnot et al., 2014; EFSA, 2011; Health Canada, 2013). These negative data were supported by structure–activity relationship data, where no structural alerts for genotoxicity were identified and a lack of suitable analogs were available for use in read‐across (US EPA, 2014). The overall weight of evidence (WOE) indicates that TBBPA does not exert genotoxic or mutagenic effects.

EFSA (2011) and Health Canada (2013) also assessed studies to investigate the potential carcinogenicity of TBBPA. At the time of these reports, no long‐term carcinogenicity data were available for TBBPA. Based upon the WOE that TBBPA was non‐genotoxic in vitro (EFSA, 2011; EU, 2006) and that there was no significant evidence of carcinogenic potential in repeat dose toxicity tests, EFSA (2011) concluded that TBBPA was not likely a carcinogen.

One study reported non‐malignant tumors in rats in response to oral TBBPA administration, including non‐dose‐responsive transitional cell papillomas in the urinary bladder that did not progress to malignancy, and thyroid follicular adenomas (Imai et al., 2009, as cited in EFSA, 2011). Colnot et al. (2014) discuss the available data and concluded that thyroid tumors are unsuitable for use in human risk assessment because of species sensitivity differences between rodents and humans. Health Canada (2013) concluded that the effect of TBBPA on thyroid hormones remains unclear and therefore utilized an MOE approach to show that current human exposures are below those that are likely to produce thyroid effects. COT (2004) discussed a lack of consistency in the available thyroid data and the potential for thyroid effects to be reversible. Additionally, neither thyroid tumors nor thyroid histopathology effects were seen in rats or mice treated in the 2‐year NTP assay (Lai et al., 2015). However, EFSA (2011) identified changes in thyroid homeostasis as the critical non‐cancer effect in their MOE analysis.

There was only one cancer bioassay identified in our literature search; the 2‐year cancer bioassay conducted by NTP (2014) in rats and mice exposed to 0, 250, 500, or 1000 mg kg^–1^ for 5 days a week via oral gavage in corn oil. These study details and results have been extensively reported elsewhere (Dunnick et al., 2015; Lai et al., 2015; NTP, 2014; US EPA, 2014; Wikoff et al., 2015, 2016). The primary tumors identified were uterine tumors (combined adenoma, adenocarcinoma, and MMMTs) in female rats (US EPA, 2014). Other tumors included testicular tumors in male rats, and hepatic tumors, hemangiomas/hemangiosarcomas, and intestinal tumors in male mice (US EPA, 2014). The Cancer Assessment Review Committee of the US EPA determined TBBPA as “likely to be carcinogenic to humans” based on the female rat uterine tumors and the male mice hemangiomas/hemangiosarcomas, and concluded there were no mutagenicity concerns associated with cancer development (US EPA, 2014).

NTP (2014) reached the following conclusions regarding each of these tumor types:

- testicular adenomas in male rats: “equivocal evidence of carcinogenic activity;”
- uterine epithelial tumors in female rats: “clear evidence of carcinogenic activity;”
- hepatoblastomas in male mice: “some evidence of carcinogenic activity;”
- intestinal tumors and hemangiosarcomas: “may have been related to chemical administration.”

### 3.4 Tetrabromobisphenol A uterine cancer mode of action and weight of evidence analysis

The US EPA (2005) guidelines for cancer risk assessment state that the MOA should be evaluated in determining the quantitative approach for dose–response assessment from positive human or experimental animal tumor data. This evaluation is accomplished by proposing an MOA by identification of the key events, where data on these key events include available in vivo, in vitro, and mechanistic studies. These studies are then evaluated relative to the modified Bradford Hill criteria, including strength, consistency, specificity of the association between the key event(s) and tumor outcomes, as well as consideration of the consistency of the dose–response and temporal relationship between the key event and tumors, biological plausibility of the proposed MOA, and coherence of the overall database (Meek, Palermo, Bachman, North, & Lewis, 2014). When sufficient data are available, a biologically based dose–response model is the preferred method for low‐dose extrapolation. In the absence of such data, US EPA (2005) and other groups such as OEHHA (2013) usually conduct a low‐dose extrapolation with a linear model if the chemical acts via a direct DNA‐reactive MOA or if the MOA is not known (non‐threshold), or via a threshold model based on one or more combinations of relevant tumors for a non‐DNA‐reactive MOA. However, in practice, evidence for a non‐DNA‐reactive MOA has not been sufficient for US EPA to move away from linear assessments most of the time, and a full analysis of the MOA is typically required to justify a non‐linear approach. The guideline states: “A nonlinear approach should be selected when there are sufficient data to ascertain the mode of action and conclude that it is not linear at low doses and the agent does not demonstrate mutagenic or other activity consistent with linearity at low doses” (US EPA, 2005). Other regulatory groups often rely on an MOE approach for cancer evaluation. However, many of these groups support the use of the best available science, including consideration of MOA, in their assessments.

An abbreviated MOA and WOE analysis was previously applied by Wikoff et al. (2016) to inform the quantitative approach for derivation of a cancer risk value. In the NTP 2‐year TBBPA bioassay, and as evaluated by Wikoff et al. (2015), uterine tumors in rats were identified as the most appropriate endpoint for use in derivation of a cancer toxicity value. Based on the considerable amount of evidence that TBBPA is not mutagenic, a non‐linear MOA was postulated for TBBPA‐induced uterine tumors based on interference with estrogen metabolism, as discussed by several authors (Borghoff, Wikoff, Harvey, & Haws, 2016; Dunnick et al., 2015; Hall et al., 2017; Harvey et al., 2015; Lai et al., 2015; Sanders et al., 2016; Wikoff et al., 2015), most comprehensively by Wikoff et al. (2016). The interference with estrogen is not thought to involve TBBPA binding directly to the estrogen receptor (ER). The weak affinity for the ER and other in vitro and in vivo studies suggests that TBBPA is not estrogenic (Colnot et al., 2014; Lai et al., 2015; Wikoff et al., 2016). Estrogenic effects of TBBPA are unclear as both negative and positive findings are reported in the literature, but the low TBBPA binding affinity to the ER suggests that TBBPA is not directly interacting with this receptor (Lai et al., 2015). Instead, interference with estrogen metabolism via competition for shared biotransformation pathways (glucuronidation and sulfation) is a plausible mechanism (Lai et al., 2015).

**Figure 1:**
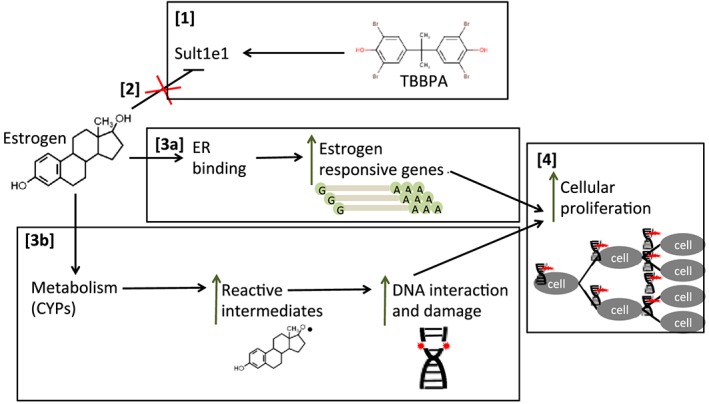
Diagram of postulated mode of action for TBBPA‐induced uterine tumors. (1) TBBPA binds to estrogen sulfotransferase (sult1e1); (2) estrogen sulfation pathway is inhibited; (3a) bioavailable estrogen can bind the ER, which translocates to the nucleus and leads to increased expression of estrogen‐responsive genes, (3b) alternative estrogen metabolic pathways (such as CYPs) can generate reactive intermediates that can interact with DNA and cause DNA damage; (4) estrogen‐responsive genes contribute to cellular proliferation of cells, some of which have increased DNA damage and gene mutations. CYPs, cytochrome P450s; ER, estrogen receptor; TBBPA, tetrabromobisphenol A [Colour figure can be viewed at http://wileyonlinelibrary.com]

Wikoff et al. (2016) proposed an adverse outcome pathway and presented data for an MOA based on a number of key events, including a WOE analysis for TBBPA‐induced uterine cancer (Figure 1; adapted from Wikoff et al., 2016). The proposed key events, starting with the molecular initiating event, are the following: (1) TBBPA binds to estrogen sulfotransferase (sult1e1), which inhibits the estrogen sulfation pathway; (2) this inhibition of estrogen sulfation leads to increased estrogen bioavailability; (3a) increased estrogen leads to increased expression of estrogen‐responsive genes, (3b) alternative estrogen metabolic pathways are activated causing generation of reactive quinones and other reactive species that can interact with DNA and cause damage and (3c) increased estrogen has the potential for disruption of the hormonal balance (and altered endocrine signaling); (4) increases in estrogen‐responsive genes contribute to cellular proliferation of cells, which may have increased DNA damage and p53 mutations; and (5) increased proliferation leads to hyperplasia of cells causing the adverse outcome (uterine tumors). These key events and supporting data are extensively discussed in Wikoff et al. (2016), and so are only briefly described below.

1. TBBPA binds to estrogen sulfotransferase (sult1e1), which inhibits the estrogen sulfation pathway.

Toxicokinetic evidence exists that shows TBBPA utilizes the same sulfation metabolic pathway as estrogen (sult1e1). TBBPA metabolites in humans include TBBPA sulfate (Schauer et al., 2006, as cited in Health Canada, 2013; Ho et al., 2017). Computational modeling and quantitative structure–activity relationship analysis suggest that TBBPA is structurally able to inhibit sulfotransferase (Gosavi, Knudsen, Birnbaum, & Pedersen, 2013; Wikoff et al., 2016). Additionally, in vitro IC_50_s for TBBPA inhibition of estrogen sulfotransferase ranges from 12 to 33 nm (Hamers et al., 2006, as cited by Borghoff et al., 2016; Gosavi et al., 2013; Kester et al., 2002; Wikoff et al., 2016). Thus, when high doses of TBBPA produce high plasma concentrations of TBBPA, the IC_50_ for sulfotransferase is surpassed and saturation can occur. For example, rat in vivo studies show that TBBPA doses as low as 50 mg kg^–1^ result in plasma concentrations (1478 nm TBBPA) well above the reported IC_50_ values (Borghoff et al., 2016; Wikoff et al., 2016).

Taken together with the in vitro data, inhibition of sulfotransferase activity is a plausible molecular initiating event in the MOA for TBBPA‐induced uterine cancer (Wikoff et al., 2016). However, more data are required to support this key event, as target tissue dosimetry and temporal relationships are required to determine if TBBPA inhibits sulfotransferase in the uterus (Osimitz, Dourson, Hayes, & Kacew, 2014).

- Inhibition of estrogen sulfation leads to increased estrogen bioavailability.

The binding of estrogen to estrogen sulfotransferase (sult1e1) leads to its biotransformation by conferring a sulfate group. When TBBPA interferes in this pathway, estrogen is not biotransformed, meaning more estrogen should be bioavailable systemically. This bioavailable estrogen could result in increased ER activation, metabolic switching to an alternative estrogen metabolic pathway, or imbalance of the estrogen/progesterone ratio that has been implicated in other tumor types (mammary, prostate) (Lai et al., 2015). However, there are currently no data on TBBPA modification of estrogen/progesterone ratios (Lai et al., 2015). Alternatively, the loss of estrogen sulfotransferase might result in increased plasma estrogen levels that are implicated in the development of estrogen‐dependent human endometrial cancer (Cornel et al., 2017).

There is a paucity of data investigating TBBPA exposure resulting in increased estrogen bioavailability, although theoretically, competition for sulfation of estrogen would reduce estrogen–sulfate conjugates, resulting in bioavailable estrogen able to bind to the ER (sulfated estrogens are not able to bind the ER) (Fu et al., 2011). This increased bioavailable estrogen could also shift the estrogen metabolic pathway to alternatives that can result in the generation of reactive species (Wikoff et al., 2016). However, Sanders et al. (2016) reported unchanged estrogen serum levels following five daily gavage doses of TBBPA at 250 mg kg^–1^, although they note that the duration of exposure might have been insufficient to produce changes and that use of serum estrogen levels serve as a poor proxy for endometrium estrogen levels.

While this step is biologically plausible, more data are needed for a definitive conclusion.

- (a) Increased estrogen leads to increased expression of estrogen‐responsive genes; (b) alternative estrogen metabolism causing generation of reactive quinones can interact with DNA; and (c) increased estrogen has the potential for disruption of the hormonal balance (and altered endocrine signaling).

Wikoff et al. (2016) discuss evidence related to increased estrogen and TBBPA‐induced increases in estrogen‐responsive genes in tissues other than the uterus. Since the time of the Wikoff publication, an additional study was published that investigated changes in estrogen concentration and gene expression in response to TBBPA. In a repeat‐dose oral gavage study, adult female Wistar Hans rats were treated with vehicle or TBBPA (250 mg kg^–1^ day^–1^) for five consecutive days to investigate the role of estrogen homeostasis in the MOA of TBBPA (Sanders et al., 2016). In tissue samples taken 24 hours after the 5 day treatment, thyroxine serum levels were decreased but serum estrogen levels were unchanged. While estrogen levels were not measured in the uterus, there were changes in the expression of genes in the uterus that are markers of cell division/growth and metabolism of TBBPA/estrogen/thyroid hormones. The gene expression changes in both the proximal and distal sections of the uterus with the greatest significance included genes involved with metabolism and hormone binding*,* including significantly increased levels of ERα and ERβ (Sanders et al., 2016). These data partially support an increase in estrogen‐responsive genes from TBBPA exposure; however, more data are needed to show that this directly results from increased bioavailable estrogen, and more data are need to identify these changes specific to uterine tissues.

Wikoff et al. (2016) discuss estrogen homeostasis as a balance of various metabolic pathways. Once one pathway is saturated, alternative estrogen metabolism pathways (other than sulfation) may compensate. One of these pathways, the catechol estrogen pathway results in the oxidation of catechol estrogens with reactive quinone intermediates. These reactive quinones can interact with DNA and have been implicated in some cancers (Wikoff et al., 2016). For example, these intermediates could be leading to DNA interactions that could contribute to or selectively increase the proliferation of altered genes, such as the tumor suppressor p53 gene.

Finally, there is a potential contribution of altered endocrine signaling via hormonal imbalance. Increased estrogen levels have the potential to modify the estrogen/progesterone ratio, and this imbalance has been implicated in other tumor types (mammary, prostate, estrogen‐dependent human endometrial cancer) (Cornel et al., 2017; Lai et al., 2015). However, there are currently no data on TBBPA modification of estrogen/progesterone ratios (Lai et al., 2015).

- Increases in estrogen‐responsive genes contribute to cellular proliferation of cells, which may have increased DNA damage and p53 mutations.

Cellular proliferation is a critical component of hyperplasia leading to tumor formation. It is well established that estrogen binding to the ER can lead to cellular proliferation as well as induction of genes related to cell cycle regulation (Sanders et al., 2016). In the NTP (2014) bioassay, there was a clear dose–response with increased uterine adenocarcinomas/adenoma at each increased TBBPA dose; however, data are lacking to confirm temporal associations specifically between increased estrogen serum levels and incidence of cellular proliferation in uterine tissues (Lai et al., 2015).

High doses of TBBPA may in part promote uterine tumors in rats by promoting growth of cells with pre‐existing mutations in the p53 tumor suppressor gene driven by increased estrogen‐dependent cellular proliferation, or through selective proliferation of these mutations caused by reactive quinone intermediates (Lai et al., 2015; NTP, 2014). Additionally, as noted above, TBBPA has low affinity for the ER and so is not likely acting directly on the ER itself. This is plausible as significantly increased p53 mutations were identified in tumors in the NTP study, but as TBBPA is non‐mutagenic, TBBPA itself is not likely directly causing the p53 mutations (Lai et al., 2015). The mechanism of p53 mutation has been previously implicated in cancer development, including human endometrial cancers (Harvey et al., 2015; Wikoff et al., 2016). Harvey et al. (2015) reported on an evaluation and analysis of TBBPA‐induced uterine carcinomas in female rats from the NTP study. Analysis using polymerase chain reaction found a high rate of p53 mutations suggesting that uterine carcinogenesis might be partially p53 dependent (Harvey et al., 2015). In this analysis, the TBBPA‐treated samples included Wistar Han rat uterine carcinomas from all dose groups combined (250, 500, and 1000 mg kg^–1^), thus no p53 mutation dose–response data are available. Of interest, the analysis did not include the MMMTs. While these data support the proposed key event, more data are needed, specifically dose–response data for p53 mutations and increased proliferation in response to TBBPA, to confirm this.

- Increased proliferation leads to hyperplasia of cells causing the adverse outcome (uterine tumors).

Hyperplasia resulting from cellular proliferation is a well‐known precursor effect related to the development of tumors, and is associated with increased estrogen levels in humans (Sanders et al., 2016). As noted, by Wikoff, both preneoplastic and non‐neoplastic hyperplasia occurred in the NTP study. Atypical endometrial hyperplasia was seen in the NTP 2‐year assay and was significantly increased above control at all dose levels; however, it was only identified via the longitudinal inspection, but not the transverse (Wikoff et al., 2016). While there was not a strict dose–response (250 mg kg^–1^ day^–1^ = 26% incidence; 500 mg kg^–1^ day^–1^ = 22% incidence; 1000 mg kg^–1^ day^–1^ = 26% incidence), preneoplastic lesions are precursors to tumor formation (Wikoff et al., 2016). Additionally, as stated above, a high incidence of p53 mutations (68%) (compared to spontaneous uterine carcinomas at 20%) was identified in the uterine carcinogenesis (Harvey et al., 2015).

Finally, the adverse outcome, significantly increased incidence of uterine tumors (adenomas, adenocarcinomas, and MMMTs), was seen with increasing dose in the NTP (2014) 2‐year assay.

#### 3.4.1 Weight of evidence

A human relevance and concordance analysis of the postulated MOA was conducted by Wikoff et al. (2016), and suggests that given the available data, the proposed MOA is plausible for the development of uterine tumors. Wikoff et al. (2016) conclude this is a plausible mechanism in humans qualitatively, but may be quantitatively excluded based on kinetic/dynamic factors between humans and rats. Given some of the data gaps associated with this MOA, we have given the greatest weight to the non‐mutagenic threshold MOA, as multiple lines of evidence support that the MOA identified is non‐mutagenic. This is seen in a number of tests showing negative mutagenicity results, which are supported by the recent NTP findings of a negative micronucleus test and two negative *Salmonella* tests. Finally, the specificity of uterine tumors to the uterine tissue only (as opposed to systemically developed tumors in multiple organs and tissues) supports the non‐mutagenic assertion (Lai et al., 2015).

Thus, while we conclude that the Wikoff et al. (2016) analysis was adequate to establish the postulated MOA, the additional information we cite is further supportive of this non‐mutagenic threshold MOA, and leads us to propose an NSRL based on the threshold approach of US EPA (2005). However, a more robust and transparent analysis of the modified Bradford Hill criteria for this MOA would be helpful. Particularly useful in this instance would be a quantitative WOE ranking, as recently proposed by Becker et al. (2017).

## 4 DERIVATION OF THE NO‐SIGNIFICANT‐RISK‐LEVEL

### 4.1 Choice of critical effect and benchmark dose lower limit analysis for point of departure

After an updated evaluation of the available carcinogenicity literature for TBBPA, we agree with the choice of Wikoff et al. (2015) that uterine tumors (adenomas, adenocarcinomas, and MMMTs combined) are the most appropriate cancer endpoint, and they were therefore chosen as the critical effect for derivation of the NSRL (Table 3). However, it is worth noting that high doses were needed to induce tumor formation, and the available evidence before the NTP assay suggested TBBPA was not carcinogenic. In addition, similar tumors were not seen in mice. Future studies can be conducted to evaluate the relevance of these tumors to humans.

**Table 3:** Dose–response and dose‐adjustment of cancer effects (tumors) and precursor effects (hyperplasia) from the NTP (2014) assay for use in benchmark dose analysis

Dose, mg kg^–1^ (NTP, 2014)	Duration‐adjusted dose	Hyperplasia response: Residual longitudinal review; endometrium, hyperplasia, atypical	Tumor response: Uterus original and residual longitudinal reviews (combined); adenoma, adenocarcinoma, or MMMT (combined)
0	0	2	6
250	180	13	11
500	360	11	16
1000	710	13	19

MMMT, malignant mixed Müllerian tumor.

Uterine tumors in female rats were chosen as the critical cancer effect for derivation of a cancer risk value. In looking at the other tumor types, the testicular adenomas in male rats were considered “equivocal” and occurred at low incidence in the two highest doses (500 mg kg^–1^, 1/50 incidence; 1000 mg kg^–1^, 13/50 incidence), and as such, were not a reliable choice for the critical effect. The hepatoblastomas in male mice had “some evidence” for carcinogenicity (250 mg kg^–1^, 2/50 incidence; 500 mg kg^–1^, 11/50 incidence; 1000 mg kg^–1^, 8/50 incidence) with a significant effect in the 500 mg kg^–1^ dose. NTP (2014) considered this as “some evidence” because after combining incidences of hepatocellular carcinomas and hepatoblastomas, there was only a significant effect at 250 mg kg^–1^. Additionally, there was no trend across doses (dose–response), and this was informed by the historical incidence of these tumor types as spontaneous and not related to chemical administration. Therefore, these tumors were not considered for use as the critical effect. The uterine epithelial tumors in female rats were the only tumor type classified as “clear evidence” and occurred with the highest incidence (0 mg kg^–1^, 6/50 incidence; 250 mg kg^–1^, 11/50 incidence; 500 mg kg^–1^, 16/50 incidence; 1000 mg kg^–1^, 19/50 incidence). Therefore, the uterine tumors were the best choice for the critical effect in derivation of a cancer risk value.

In line with the Wikoff et al. (2015) assessment, we modeled the incidence of combined uterine adenomas, adenocarcinomas, and MMMTs observed in female rats (NTP, 2014). While we agree with Wikoff et al. (2015) on the choice of critical effect, the application of the BMD approach, use of BMDL_10_ and allometric adjustment of the POD to an HED, we had the benefit of additional literature that allowed us to support a non‐mutagenic, threshold MOA and the determination of an RfD_cancer_ through the application of UFs to the POD analogous to an RfD or TDI approach (US EPA, 2005).

Specifically, our conclusion is supported by Wikoff et al. (2016) who suggest that the linear cancer slope factor approach is inappropriate for a non‐mutagenic chemical, and they indicate that a threshold approach based on a non‐mutagenic MOA is most appropriate. In fact, the derivation of an oral slope factor by these authors was likely due to uncertainty in regulatory policy that suggests an MOA is needed to move away from a linear assessment. However, as noted above in Section 3.4, according to US EPA “sufficient data to ascertain the mode of action” is needed along with a conclusion of non‐linearity at low doses coupled with non‐mutagenicity data (US EPA, 2005). This conclusion of a non‐linear MOA for TBBPA is supported in the extant literature as cited by Wikoff et al. (2015), and further supported by Sanders et al. (2016) and Lai et al. (2015). Thus, we selected a non‐linear approach, as there are sufficient data to conclude that the MOA is not linear at low doses and TBBPA is clearly non‐mutagenic. In addition, the specificity of the tumor response to specific tissues further supports a threshold approach as the most scientifically credible to develop an RfD_cancer_.

**Figure 2:**
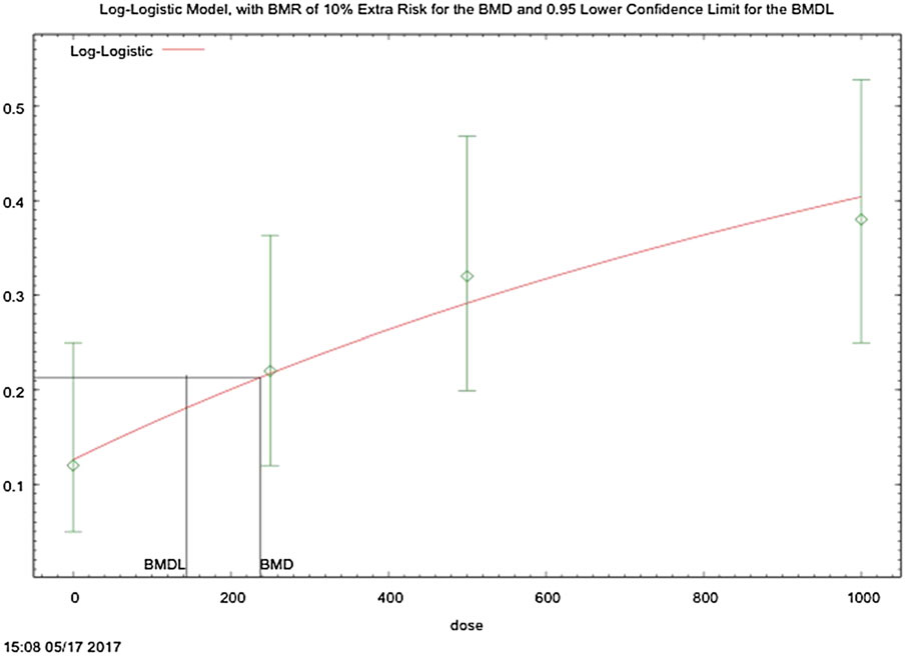
Log‐logistic modeling results of uterine cancer (adenoma, adenocarcinoma, or malignant mixed Müllerian tumors, combined) in female rats from NTP (2014). Dose in mg kg^–1^ is presented on the *x*‐axis and probability of response is presented on the *y*‐axis. Benchmark dose (BMD) and the 95% lower confidence limit (BMDL) representing 10% extra risk is shown with the black line [Colour figure can be viewed at http://wileyonlinelibrary.com]

The results of the BMD analysis on adenoma, adenocarcinoma, or MMMT (combined) incidence in relation to TBBPA exposure are shown in Table 4. The log‐logistic model (Figure 2) best fits the data based on all quantitative fit criteria: *P* value (0.85), scaled residuals (0.042) at the dose with the response closest to the BMR, good visual fit, BMD/BMDL ratio less than 2 and lowest AIC (222.8), resulting in a dose‐adjusted BMD_10_ of 169 mg kg^–1^ day^–1^ corresponding to the BMDL_10_ of 103 mg kg^–1^ day^–1^. This model provides a similar BMD to that from the multistage model (i.e., the model chosen by Wikoff et al., 2015), but the log‐logistic model results better fit the data, particularly in the dose region of interest (at the BMR).

**Table 4:** BMD models examining the relationship between TBBPA exposure^a^ and uterine cancer incidence (adenoma, adenocarcinoma, or malignant mixed Müllerian tumors, combined) in female rats from NTP (2014)

Model	*P* value	Scaled residual at dose	Visual fit	Ratio BMD/BMDL	AIC	BMD_10_ (rounded)	BMDL_10_ (rounded)
Gamma	0.75	0.14	Good	1.5	223.1	200	130
Logistic	0.46	0.88	Acceptable	1.3	224.0	290	220
**Log‐logistic**	**0.85**	**0.042**	**Good**	**1.7**	**222.8**	**170**	**100**
LogProbit	0.32	0.89	Acceptable	1.5	224.8	320	220
Multistage (1^b^)	0.75	0.14	Good	1.5	223.1	200	130
Multistage (2^b^)	0.75	0.14	Good	1.5	223.1	200	130
Multistage (3^b^)	0.75	0.14	Good	1.5	223.1	200	130
Probit	0.49	0.84	Acceptable	1.3	223.9	280	210
Weibull	0.75	0.14	Good	1.5	223.1	200	130
Quantal‐linear	0.75	0.14	Good	1.5	223.1	200	130

AIC, Akaike information criterion; BMD, benchmark dose; BMDL, benchmark dose lower limit.

a Duration‐adjusted dose (5/7 days).

b Numbers correspond to the number of degrees of polynomial in the multistage model.

Row in **bold** indicates the best fitting model.

Atypical hyperplasia of the endometrium was also modeled as a potential precursor effect to tumor formation, but models had worse fitting than those for the tumor endpoints (i.e., all models had *P* < 0.1), possibly due to toxicity masking at the high dose. For example, Table 3 shows that the incidence of hyperplasia was not increased at doses where tumors were induced. Removal of the high‐dose data for hyperplasia marginally improved model fit, but still no model adequately fit the data as compared to the tumor endpoint (see Table 5). BMDs and BMDLs for hyperplasia were only approximately 1.5‐fold lower than that calculated from the uterine tumors (Tables 4 and 5), but carry larger uncertainty due to the apparent lack of dose–response. See Section 5.3 (“Uncertainties”) for more discussion on the hyperplasia data.

**Table 5:** BMD models examining the relationship between TBBPA exposure^a^ and atypical hyperplasia incidence in female rats from NTP (2014)

Model	*P* value	Scaled residual at dose	Visual fit	Ratio BMD/BMDL	AIC	BMD_10_ (rounded)	BMDL_10_ (rounded)
Gamma	0.06	1.5	Acceptable	1.5	134.2	130	90
Logistic	0.02	1.9	Poor	1.4	136.5	220	160
**Log‐logistic**	**0.08**	**1.4**	**Acceptable**	**1.6**	**133.7**	**120**	**70**
LogProbit	0.01	2.1	Poor	1.4	137.4	190	130
Multistage (1^b^)	0.06	1.5	Acceptable	1.5	134.2	130	90
Multistage (2^b^)	0.06	1.5	Acceptable	1.5	134.2	130	90
Multistage (3^b^)	0.06	1.5	Acceptable	1.5	134.2	130	90
Probit	0.02	1.9	Poor	1.4	136.3	210	150
Weibull	0.06	1.5	Acceptable	1.5	134.2	130	90
Quantal‐linear	0.06	1.5	Acceptable	1.5	134.2	130	90

AIC, Akaike information criterion; BMD, benchmark dose; BMDL, benchmark dose lower limit.

a Duration‐adjusted dose (5/7 days).

b Numbers correspond to the number of degrees of polynomial in the multistage model.

Row in **bold** indicates the best fitting model.

The resulting duration‐adjusted BMDL_10_ of 103 mg kg^–1^ day^–1^, based on uterine tumors, was adjusted to HED of 25.6 mg kg^–1^ day^–1^ using allometric scaling (Equation 1; [25.6 mg kg^–1^ day^–1^ = 103 mg kg^–1^ day^–1^ × (0.268 kg/70 kg)^1/4^]). (As noted previously, the choice of default body weight [between females at 58 kg and males at 70 kg] does not significantly change the resulting HED [26.9 mg kg^–1^ day^–1^ vs. 25.6 mg kg^–1^ day^–1^, respectively]. For this and the reasons listed earlier, we have used the default body weight of 70 kg.) The HED would be 17.4 mg kg^–1^ day^–1^ if using the lowest BMDL of 70 mg kg^–1^ day^–1^ from the atypical hyperplasia data (Table 5). See Section 5.3 (“Uncertainties”) for a discussion on the relevance of the hyperplasia endpoint.

### 4.2 Uncertainty factors

UFs were applied to the BMDL_10[HED]_ to derive an RfD_cancer_ of 0.3 mg kg^–1^ day^–1^ using Equation (2) (0.26 mg kg^–1^ day^–1^ = 25.6 mg kg^–1^ day^–1^/(10 × 3 × 3 × 1 × 1 = 100).

- UF that addresses interindividual variability (UF_H_) (also referred to as intraspecies variability) accounts for toxicokinetic and toxicodynamic variation across humans and is intended to protect sensitive subpopulations. Unless a study is conducted in a sensitive human population or there are data on human variability in response, the default for the UF_H_ is 10. Given the lack of available data to move away from the default, we recommend the application of a 10‐fold factor.
- UF for interspecies extrapolation (UF_A_) (also referred to as animal‐to‐human extrapolation) accounts for the translation of data from experimental animals to humans, specifically the toxicokinetic and toxicodynamic variation between species. Because we adjusted the POD to a HED, this is presumed to account for the toxicokinetic differences across species (Renwick, 1999). Therefore, a reduced factor of one‐half the power of 10 (~3‐fold) should be applied to account for the toxicodynamic differences between species (Renwick, 1999).
- UF for use of a lowest observed adverse effect level and extrapolation to a NOAEL (UF_L_) is not needed, as a BMD analysis was conducted. Therefore, a factor of 1 is applied. Additionally, the UF for extrapolation of a subchronic critical study to a chronic exposure (UF_S_) is not necessary, as a 2‐year cancer bioassay was selected as the critical study. Therefore, a factor of 1 is applied.
- UF for database completeness (UF_D_) represents a judgment on the quantity and quality of the toxicology information available, particularly in the number of experimental species tested and whether or not developmental and reproductive studies are available. TBBPA has an adequate toxicological database, in this regard, to assess the toxicological outcomes and potential adverse effects from exposure. However, this factor has also been utilized on occasion to account for effects that are not addressed directly by the POD, or other data gaps (e.g., neurological). In pharmaceutical risk assessment, additional scientific judgment associated with the data set can be accounted for under this UF (Sussman et al., 2016). While the availability of the NTP 2‐year comprehensive cancer bioassay is sufficient to inform the database for cancer and while there is a lack of evidence suggesting TBBPA is highly carcinogenic, we opted to include an additional factor of 3 given the uncertainty associated with modeling the tumor precursor data (hyperplasia) due to potential toxicity masking and for the decision to model an overt tumor endpoint as opposed to the precursor. As more cancer assessments move away from the default linear approach with the incorporation of more information on MOA, we envision the database UF encompassing these types of adjustments as a place to account for additional uncertainties.

In total, we recommend the application of a composite UF of 100 (3 × 3 × 10) to protect for uncertainties in the database and extrapolations.

Therefore, for the derivation of the oral NSRL, we first divide the BMDL_10[HED]_ of 26 mg kg^–1^ day^–1^ by 100 to derive a cancer safe dose of 0.26 mg kg^–1^ day^–1^ (rounded to correct significant figures = 0.3 mg kg^–1^ day^–1^). Based on the default human body weight of 70 kg, and using Equation (3) (0.26 mg kg^–1^ day^–1^ × 70 kg = 18 mg day^–1^), the oral NSRL is rounded to 20 mg day^–1^.

There were not enough published data identified to derive an inhalation NSRL. There was at least one DNEL derived for inhalation exposure (EHCA, 2017); however, the studies that those values were based on were not publically available, and the relevance to cancer development from inhalation exposure remains uncharacterized.

## 5 DISCUSSION

### 5.1 Comparison of no‐significant‐risk‐level to risk‐specific dose published by Wikoff et al. (2015)

An NSRL of 20 mg day^–1^ was adapted from an RfD_cancer_ of 0.3 mg kg^–1^ day^–1^ based on a threshold MOA for uterine cancer development in the NTP (2014) bioassay. The NSRL value (20 mg day^–1^) is ~90‐fold higher than the cancer slope factor adjusted to an NSRL derived by Wikoff et al. (2015) for 10^–5^ risk for the same tumor data (the risk level assigned by the NSRL) (0.0032 mg kg^–1^ day^–1^ × 70 kg = 0.22 mg day^–1^). This difference reflects the use of a threshold approach instead of a slope factor for low‐dose extrapolation, and slight differences in the BMDL due to model selection. Table 4 shows the various BMD model outputs for the uterine tumor data. While the output of our models appears to align with those of Wikoff et al. (2015), we chose a different model for a POD based on an evaluation of multiple parameters (*P* value, scaled residuals, visual fit, ratio of BMD to BMDL, and AIC). This difference in model selection accounts for a ~20% difference in the chosen points of departure (126.6 mg kg^–1^ day^–1^ chosen by Wikoff and colleagues vs. 103 mg kg^–1^ day^–1^ chosen for this assessment).

The NSRL proposed here of 20 mg day^–1^, however, is within an order of magnitude of the Wikoff et al. (2015) RfD of 0.6 mg kg^–1^ day^–1^ for uterine hyperplasia (0.6 mg kg^–1^ day^–1^ × 70 kg = 42 mg day^–1^). As some types of uterine hyperplasia are considered an upstream precursor to uterine cancer, the alignment of these values makes sense biologically. While protection from precursor effects is typically anticipated to protect from the downstream cancer effect, in this case our RfD_cancer_ is lower than the RfD for the precursor hyperplasia. The fact that our value is lower than that of a precursor supports our choice to not model the hyperplasia precursor due to uncertainties in the data as BMD models were not able to fit the data adequately (*P* < 0.1), even when the responses at the highest dose were dropped from the model (an approach consistent with US EPA guidance; US EPA, 2012). Additional differences between these RfDs stem from the application of different UFs (we applied 100 to the tumor endpoint and 30 for the hyperplasia). See Section 5.3 (“Uncertainties”) for a discussion on the relevance of the hyperplasia endpoint.

### 5.2 Comparison of RfD_cancer_ to available risk values

**Figure 3:**
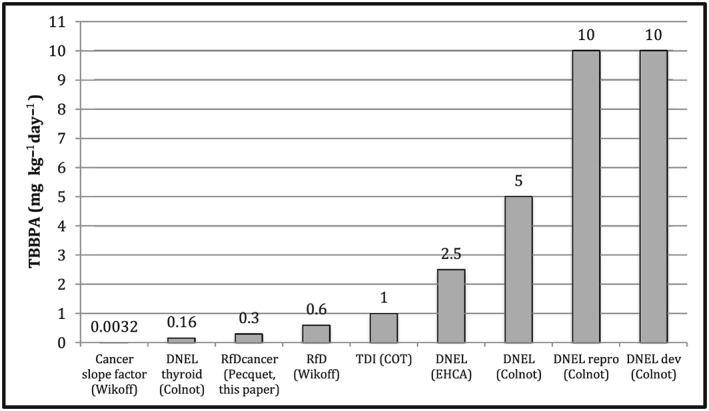
Comparison of available cancer and non‐cancer risk values for TBBPA. DNEL, derived no effect level; DNEL_dev_, derived no effect level non‐cancer developmental effects; DNEL_repro_, derived no effect level non‐cancer reproductive effects; RfD, reference dose; RfD_cancer_, reference dose for cancer effects; TBBPA, tetrabromobisphenol A; TDI, tolerable daily intake. References include: Wikoff et al. (2015); COT (2004); Colnot et al. (2014); ECHA (2017)

A comparison was made between the RfD_cancer_ derived here and other available risk values (see Table 2; Figure 3). The derived RfD_cancer_ (0.3 mg kg^–1^ day^–1^) falls appropriately in respect to the biology on the risk value continuum as shown in Figure 3. As expected, DNELs for non‐cancer reproductive and developmental effects (DNEL_repro_ and DNEL_dev_, both = 10 mg kg^–1^ day^–1^) and DNELs for non‐cancer no‐effect levels (5 and 2.5 mg kg^–1^ day^–1^) are higher than the derived RfD_cancer_ by ~8–33‐fold. The TDI, which was also derived for a non‐cancer no‐effect level (1 mg kg^–1^ day^–1^), is ~3‐fold higher than the RfD_cancer_, but is within an order of magnitude of this value. This makes biological sense given the threshold MOA for uterine tumor formation. The RfD for uterine hyperplasia (0.6 mg kg^–1^ day^–1^) is slightly above the RfD_cancer_, but well within an order of magnitude. This is expected and makes biological sense given that uterine hyperplasia is a potential precursor effect to uterine tumors, although one would expect an RfD for a precursor effect to be lower than that for the apical tumor effect. Finally, the DNEL for thyroid effects (0.16 mg kg^–1^ day^–1^) is lower than all other available non‐cancer values. However, as noted above in Section 3.3.1, there is a large amount of uncertainty associated with the thyroid endpoint (species sensitivity differences between rodents and humans, lack of consistency in the available thyroid data, potential for the effect to be reversible, and fact that neither thyroid tumors nor thyroid histopathology effects were seen in rats or mice treated in the 2‐year NTP assay). Finally, the cancer slope factor (0.0032 mg kg^–1^ day^–1^) is significantly lower than all other available risk values (from 50‐ to ~3000‐fold lower). Typically, the expectation is for cancer risk values to be lower than that for non‐cancer, under a no‐threshold assumption. However, given the evidence for a threshold MOA for the uterine tumors, the cancer slope factor is likely highly conservative and not biologically appropriate (~100‐fold lower than the RfD_cancer_) (Bevan & Harrison, 2017).

### 5.3 Uncertainties

Our choice was to develop an RfC_cancer_ for the tumor endpoint as opposed to an RfD based on the hyperplasia precursor. The main reason for this choice was that all of US EPA's standard BMD models failed the standard US EPA criteria for *P* > 0.1 when all doses were considered for the hyperplasia. This might have been due to toxicity masking at the high dose, where the incidence of hyperplasia was the same as the lowest dose (which was 40‐fold lower) and potentially “hidden” by tumor formation. After dropping the high dose and rerunning the models, all models again failed the standard US EPA criteria for *P* > 0.1, but in this case, several models had *P* > 0.05 to *P* < 0.1. US EPA accepts *P* > 0.05 for multistage models; however, other aspects of model fit were evaluated alongside the *P* value (see Section 2.2 Methods). Among these criteria, visual fit for the hyperplasia data was “adequate” or “poor” (while many models were “good” for the tumor endpoints), and the need to drop the high‐dose data was qualitatively concerning. BMDs varied among these models from 120 to 210 mg kg^–1^ day^–1^ (Table 5), and BMDLs ranged from 70 to 160 mg kg^–1^ day^–1^ (Table 5). In contrast to these hyperplasia models, the tumor modeling was well supported at all doses (Table 4), where *P* values were uniformly acceptable, and several models could be used based on the POD.

While the idea is not to limit the modeling outcomes based solely on prescriptive model fit, our decision to rule out the hyperplasia precursor was ultimately due to the uncertainty in the modeling of this effect and lack of apparent dose–response (due to its possible toxicity masking at higher doses). Such masking makes modeling more uncertain because some of the data need to be disregarded (in this case the high‐dose data were dropped), which is not preferable. The availability of a non‐cancer RfD already derived for hyperplasia (Wikoff et al., 2015) and the proximity of that RfD to our RfD_cancer_ for the uterine tumor outcome is reassuring, and further supports the threshold mechanism.

Thompson et al. (2016) used a precursor effect to derive an NSRL for titanium dioxide; however, there are extensive MOA data for this chemical and an available and fully vetted adverse outcome pathway for this tumor endpoint. Their ability to use a defined precursor likely stems from the vast amount of available data. For example, in Thompson et al. (2016), they supply this quote from US EPA: “When good quality precursor data are available and are clearly tied to the mode of action of the compound of interest, models that include both tumors and their precursors may be advantageous for deriving a POD.”

The use of an additional factor of 3 in the database UF for our cancer RfD relates to the uncertainty in modeling the hyperplasia precursor as a critical effect and the use of the overt tumor endpoint, not to the overall database for TBBPA itself. This factor offers a more conservative (health protective) safe dose and can be seen to bridge the gap between the 1.5‐fold lower BMDLs for hyperplasia. In fact, an NSRL based on the precursor would be higher than the NSRL for the tumor endpoint. (If we use the lowest hyperplasia HED of 17 mg kg^–1^ day^–1^ and apply a 30‐fold UF, the resulting value is 0.58 mg kg^–1^ day^–1^, equating to an NSRL of 41 mg day^–1^, which is double the value derived for the tumor effect.) As this is impossible mechanistically and biologically (that tumors occur at lower doses than the precursor hyperplasia), this renews our confidence that the hyperplasia data are a poor choice as compared to the tumor data for the critical effect. While the addition of the UF_D_ of 3 for tumors but not hyperplasia drives the tumor RfD below that for hyperplasia, the proximity of the PODs (70 mg kg^–1^ for hyperplasia compared to 103 mg kg^–1^ for tumors) and the HEDs (25.6 mg kg^–1^ for tumors compared to 17 mg kg^–1^ for hyperplasia) suggests that these endpoints are not that far apart in relation to dose. Additional uncertainty is associated with the hyperplasia data (toxicity masking, high‐dose dropping, poorer model fit) that is not associated with the tumor data. Additionally, the RfDs for tumors and hyperplasia are within an order of magnitude of each other, and therefore are not considered significantly different from one another as stated by the US EPA (1993) (“the RfD is an estimate (with uncertainty spanning perhaps an order of magnitude)”). While we understand that the choice of critical effect is a scientific judgment, the resulting RfDs for hyperplasia and for tumor formation are essentially identical, suggesting that both endpoints will be protected from at the derived RfD_cancer_. Because we chose to err on the conservative side, we have chosen the lower of the two NSRLs, which is from the tumor endpoint after application of an additional UF.

We anticipate that as more cancer assessments are based on non‐linear threshold mechanisms as the basis for safe dose derivation, the UF for database completeness might expand to include uncertainties such as accounting for a precursor when the data cannot be modeled.

Uncertainties are associated with using the MMMT data combined with the uterine adenomas and adenocarcinomas because of the rarity in MMMT occurrence and the fact that a dose‐dependent trend was not reported in TBBPA‐treated rats. MMMTs are a very rare, spontaneous neoplasm in rats (Dunnick et al., 2015). Furthermore, the historical data “are limited in Wistar Han rats because few studies using this strain have been conducted” (NTP, 2014). However, a large body of evidence on the epithelial histogenesis of MMMTs and their relevance to uterine cancers was cited as reasoning to include the MMMTs (Dunnick et al., 2015). The use of a new method of examining the rat uterus (a secondary residual longitudinal review combined with the initial standard transverse review) allowed for the identification of additional tumors; the additional transverse review identified adenocarcinomas or adenomas in all female rats with MMMTs.

The MOA for uterine tumor formation needs additional validation, specifically, it would highly benefit from a comparison to the modified Bradford Hill criteria (as conducted in Meek et al., 2014) and a quantitative WOE approach (as previously demonstrated for clofibrate in Becker et al., 2017). For the MOA, in vivo data to confirm that TBBPA competes for estrogen sulfotransferases are lacking. Target tissue dosimetry and temporal relationships to determine if TBBPA inhibits sulfotransferase in the uterus are required to determine to validate this mechanism (Osimitz et al., 2014). Other uncertainties in the estrogen metabolism pathway have not been addressed, including the role of the alternative estrogen metabolism pathways, such as induction of phase I enzymes CYP1A1 and CYP1B1 (leading to reactive metabolite formation) (Sanders et al., 2016). Others reviewed the plausibility of these alternative pathways but a more in‐depth review is needed (Dunnick et al., 2015; Sanders et al., 2016; Wikoff et al., 2015).

Additionally, more data are needed to evaluate this MOA at human relevant exposure doses. Wikoff et al. (2016) and others suggest this MOA operates only at high doses where saturation of the estrogen metabolic pathway occurs. Wikoff et al. (2016) suggests extrapolation to lower doses for the protection of human health may be inappropriate given human doses are not expected to be high enough to lead to this MOA. However, we provide clear rationale that our NSRL is appropriate and as applied, is protective of the development of uterine tumors for a few reasons: (1) tumors appear to be formed only at high doses due to non‐mutagenic mechanism, and no tumors were identified in previous studies except the non‐malignant tumors (transitional cell papillomas in the urinary bladder and thyroid follicular adenomas) (Imai et al., 2009, as cited in EFSA, 2011). This suggests that the potential for carcinogenicity from TBBPA exposure is quite low, will only occur at high doses, and negates the need for low‐dose extrapolation, and (2) Wikoff et al. (2016) reports that doses of 50 mg kg^–1^ are enough to surpass the sulfotransferase IC_50_, suggesting that this mechanism could be activated at doses below those in the NTP study. However, this dose would need to be exceeded in a chronic fashion for tumor formation to occur, and the RfD_cancer_ is well below this IC_50_ (0.3 mg kg^–1^ day^–1^). Therefore, the derived RfD_cancer_ is protective of uterine tumors via a non‐threshold MOA, and low‐dose extrapolation is not necessary.

A final caveat relates to the existence of other potential MOAs. Effects on thyroid homeostasis have been seen and for non‐cancer effects have produced relatively low BMD/Ls. Studies have shown that high TBBPA concentrations in vitro inhibit thyroid hormone metabolism with an IC_50_ of 460 nm for SULT1A in human liver cytosol, and the contribution of this MOA remains unclear (Butt & Stapleton, 2013). However, there is little indication in the NTP (2014) assay that thyroid tumors result from exposure to TBBPA, as neither tumors nor histopathology were found following exposure for 2 years. Additionally, there were testicular adenomas and hepatoblastomas identified in the NTP (2014) report. It is possible that these tumor types might drive the RfD_cancer_ value lower, but as for uterine tumors, thyroid tumors would also be anticipated to be developed via a non‐mutagenic threshold MOA due to the non‐mutagenic nature of TBBPA.

## 6 CONCLUSIONS

Building off of previously published work investigating the MOA and toxicity of TBBPA (ESFA, 2011; Health Canada, 2013; Lai et al., 2015; Wikoff et al., 2015, 2016), and using the cancer results seen from the recent NTP 2‐year cancer bioassay, we have derived an NSRL for TBBPA of 20 mg day^–1^. The NSRL is based on uterine tumors (adenomas, adenocarcinomas, and MMMTs) identified in female rats exposed to TBBPA for 2 years via oral gavage. TBBPA has been shown to act through a non‐mutagenic MOA, and as such, the most appropriate approach to derivation of a cancer risk value is a threshold approach, akin to an RfD_cancer_. Using the NTP study data, we derived a BMDL_10_ POD of 103 mg kg^–1^ day^–1^ and adjusted this to a HED of 26 mg kg^–1^ day^–1^ using allometric scaling. We applied a composite adjustment factor of 100 to the POD to derive an RfD_cancer_ of 0.3 mg kg^–1^ day^–1^. Based on an average human body weight of 70 kg, the cancer safe dose was adjusted to an NSRL of 20 mg day^–1^.

## CONFLICT OF INTEREST

The authors did not report any conflict of interest. This manuscript was developed from a report on the cancer and non‐cancer toxicology of TBBPA submitted to the American Chemistry Council (ACC) under a previous contract. Additional funding was supplied from ACC and from the Risk Science Center (RSC) of the University of Cincinnati to turn the report into a manuscript. As such, before journal submission, the manuscript was reviewed by RSC affiliates and ACC. We also note the additional review and comments from the journal reviewers, which substantially enhanced this paper. Comments received by these groups were in some cases accepted and other times rejected, at the discretion of the co‐authors and based on scientific relevance. This manuscript reflects the scientific analyses and opinions of the co‐authors and not those of funding organizations.
